# Exploring Empathic Space: Correlates of Perspective Transformation Ability and Biases in Spatial Attention

**DOI:** 10.1371/journal.pone.0005864

**Published:** 2009-06-10

**Authors:** Katharine N. Thakkar, Peter Brugger, Sohee Park

**Affiliations:** 1 Department of Psychology, Vanderbilt University, Nashville, Tennessee, United States of America; 2 Department of Psychiatry, Vanderbilt University, Nashville, Tennessee, United States of America; 3 Department of Neurology, University Hospital, Zurich, Switzerland; University of Granada, Spain

## Abstract

Separate lines of research have noted recruitment of parietal cortex during tasks involving visuo-spatial processes and empathy. To explore the relationship between these two functions, a self-other perspective transformation task and a task of spatial attention (line bisection) were administered to 40 healthy participants (19 women). Performance on these tasks was examined in relation to self-reported empathy. Rightward biases in line bisection correlated positively with trait-level self-reported empathic concern, suggesting a left hemisphere mediation of this prosocial personality trait. Unexpectedly, speed of perspective taking in the self-other transformation task correlated *negatively* with empathic concern, but only in women, which we interpret in light of gender differences in empathy and strategies for egocentric mental transformations. Together, the findings partially support the commonalities in visuo-spatial attention, perspective-taking and empathy. More broadly, they shed additional light on the relationship between basic cognitive functions and complex social constructs.

## Introduction

“…we observe a man's actions and place ourselves partly but not wholly in his position; or we act, and place ourselves partly in the position of an outsider.”

T.S. Eliot [1]

Spatial metaphors are often used to describe empathy (i.e., putting oneself in another's shoes), but little work has been done to examine the empirical relationship between empathy and visuo-spatial abilities. Interestingly, there seems to be shared brain regions associated with these two functions; parietal regions have long been implicated in visuo-spatial processing [for review see 2], and recent neuroimaging work investigating neural correlates of empathic responding have also noted recruitment of parietal networks [3], [4]. The major aim of the present study was to investigate the relationship between self-reported trait empathy and indices of visuo-spatial ability that have been associated with parietal cortical functioning, specifically imagined self-other transformations and biases in spatial attention. A secondary aim was to examine this putative relationship as a function of gender, as sex differences have been reported for both empathy [5] and visuo-spatial processing [6].

One problem that impedes the scientific study of empathy is the apparent difficulty in reaching a consensus on the definition. In the most general sense, empathy refers to processes of interpreting and reacting to the experiences of others, and many researchers agree that empathy is a multifaceted construct that involves both cognitive and emotional components [see 7]. Cognitive empathy refers to a controlled process by which an individual projects himself or herself into the place of another. It is closely akin to the construct of ‘theory of mind’, attribution of mental states to oneself and others. On the other hand, emotional empathy commonly refers to the more automatic affective response to the experience of others, which can motivate concern and subsequent helping behavior. It is argued that a sense of shared interpersonal space, or self-other equivalence, is a basic prerequisite for empathy [8]. An interesting question stems from this idea: to what extent is this shared interpersonal space visuo-spatially represented? How is perspective-taking, in the abstract sense, related to visuo-spatial perspective-taking and imagined self-other transformations? Amorim [9] provides a conceptual link between social perception and understanding what another individual sees. He notes that using visual cues such as eye gaze to discern where another individual is directing attention requires the coordination of one's own perspective and the perspective of a second party.

Egocentric mental rotation refers to imagined changes in position and orientation relative to the surrounding environment. On the other hand, object-centered mental rotation involves mentally manipulating an object relative to its own reference frame. A specific subset of egocentric mental rotation tasks, in which subjects are required to mentally transform themselves into the body of another, can be used to investigate visuo-spatial self-other transformations. These tasks typically require individuals to imagine taking the position of a figure on a screen and make judgments about the location of body parts [see 10]. It has been consistently reported that response times (RTs) are longer when the position of the figure does not match the position of the subject, because he/she has to make an additional perspective transformation [e.g. 10], [11]. This finding is consistent with observations on the mental rotation of objects; RTs for mental rotation correspond to RTs for physical rotation and increase as the required degree of mental rotation increases [12].

Evidence from the neuropsychological and neuroimaging literature suggests that the specific brain structures involved in egocentric mental rotation are partially distinct from those involved with object-centered rotation. Results differ somewhat across neuroimaging studies, likely because of the particular task demands. During functional magnetic resonance imaging (fMRI), parietal cortex [13]–[15], medial prefrontal cortex [e.g. 13], premotor cortex [15], supplementary motor areas [14], and inferior frontal cortex [14], [15] have been found to be more active when individuals are asked to make judgments about an external scene from the viewpoint of an allocentric, or second-person, perspective versus a first-person perspective. Imagined rotation of a body part, usually a hand, recruits a similar network of parietal and premotor regions [e.g. 16]. Neural correlates of whole-body self-other transformations include the left frontal cortex [e.g. 11] and the temporo-parietal junction (TPJ) [11], [17]. Evidence supporting lateralized activity of the TPJ during own-body mental transformations is mixed, with some evidence supporting left hemisphere [11], and others supporting a stronger role of the right hemisphere [17]. Moreover, lesions of the TPJ have been associated with out-of-body experiences, the phenomenon of observing one's body from an apparently external viewpoint [18].

Interestingly, recent studies investigating the neural underpinnings of empathy and shared metaphoric interpersonal space have also highlighted the role of the right TPJ, especially with regards to distinguishing one's own perspective from others'. Increased right TPJ activity has been associated with adopting the perspective of another individual [e.g. 19], and has been suggested to play a major role in a sense of agency [20].

Social and emotional processes are typically thought to be mediated predominantly by the right hemisphere [see 21], however a simple right-left hemispheric distinction is an oversimplification. For example, in a sample of unilateral frontal and posterior lesion patients, both right and left frontal lesions were found to impair empathic abilities equally; but in the posterior lesion group, only those with right lesions were impaired [22]. Buck [23] hypothesizes that emotions have both individualistic and prosocial functions. Some emotions function for self preservation and others function toward preservation of the species, and he posits that they are associated with right and left hemispheres, respectively. This theory has received some empirical support. During the Wada procedure, in which one hemisphere is inactivated, changes in behavior following inactivation of the right hemisphere were consistent with a change from “selfish” to social emotions [24]. Moreover, MacLean [25] suggests that cingulate areas are associated with prosocial emotions and temporal limbic systems are associated with individualistic emotion, and in a resting state metabolism study, Gur et al., [26] found greater left lateralized metabolism in cingulate gyrus and greater right lateralized metabolism in the temporal limbic system. To date, the unique roles of left and right hemispheric contributions to empathy are thus unclear, especially compared to the far more unequivocal picture with respect to the lateralization of spatial attention.

Asymmetries in hemispheric activation can be indexed by measuring the spatial distribution of attention, according to the activation-orienting hypothesis, [27], which suggests that there is a bias to orient attention in the direction contralateral to the more activated hemisphere. This lateralized bias in attentional orienting holds whether the contralateral hemisphere is stimulated or the ipsilateral hemisphere is inhibited. Biases in the orienting of spatial attention may be assessed reliably using the line bisection task, which is commonly administered to unilateral neglect patients and healthy controls. Consistent with the activation-orienting hypothesis, individuals with lesions of the right inferior parietal or TPJ exhibit pronounced spatial neglect of the left hemifield [e.g. 28], and consequently bisect horizontal lines markedly to the right of center [29]. Similarly, healthy individuals tend to show a subtle deviation to the left in line bisection, referred to as “pseudoneglect” [for review see 30]. Most proposed mechanisms for this deviation relate to right hemisphere dominance in the control of spatial attention [31]. Although the relative hemispheric dominance indexed by the line bisection task has not been related to measures of empathy, lateral deviations have been found to be correlated with other personality measures associated with hemispheric lateralization such as trait affect [32] and magical ideation [33].

The aim of the current study was to examine the relationship between self-report measures of trait-level empathy and visuo-spatial processing, namely imagining self-other perspective transformations and biases in spatial attention, in healthy individuals. We predicted that: 1) efficiency of an imagined visuo-spatial self-other transformation would be associated with increased self-reported empathy, given its face validity and evidence for common brain regions involved in both functions; 2) higher self-report empathy scores would be associated with right hemispheric activity, and thus increased pseudoneglect (leftward bias) given the literature on right hemispheric involvement in emotional processes. We also explored whether the relationship between visuo-spatial processing and empathy would differ across genders, given reported gender differences in both visuo-spatial skills [6] and empathy [e.g. 5].

## Methods

### Participants

40 healthy subjects (19 females) were recruited by community fliers and an e-mail advertisement at the University of Zurich and the Swiss Federal Institute of Technology (Eidgenössische Technische Hochschule Zürich). Participants were screened for a history of mental illness in themselves or family members, drug use, head injury, and left-handedness according to a 13-item handedness questionnaire [34]. Subjects had a mean age of 26.1 (s.d. = 6.7 years) and 16.3 (s.d. = 2.8) years of education. Age and education did not differ significantly between males and females (age: t(38) = 1.55, p = .13; education: t(38) = 0.93, p = .36). The study protocol was approved by the Vanderbilt University Institutional Review Board and was in specific agreement with ethical and safety guidelines from the University Hospital of Zürich. Written informed consent was obtained from all subjects prior to testing, and experiments were conducted according to the Declaration of Helsinki. Subjects were compensated for their participation.

### Self-reported empathy questionnaire

The Interpersonal Reactivity Index [IRI; 35] is a 28-item empathy measure consisting of four subscales: Perspective-Taking (PT), Fantasy (FS), Empathic Concern (EC), and Personal Distress (PD), and assessed using a 5-point Likert scale (0–4). We used the German translation of the IRI by Paulus [36], without the items comprising Competence as a 5^th^ subscale. The IRI was developed using a multidimensional approach and was designed to evaluate both the cognitive and affective components of empathy. The PT subscale assesses the tendency to adopt the psychological viewpoint of others, and the FS subscale assesses the tendency to transpose oneself into the experience of a fictitious character; these two scales were designed to measure the cognitive component of empathy. The EC scale assesses “other-oriented” feelings of sympathy and concern, and the PD scale measures feelings of interpersonal anxiety in response to other people's distress. Since scores on the FS and PD scales have been associated with social dysfunction, and the PD scale was found to be negatively correlated with other empathy measures [37], we focused on the more psychometrically validated PT and EC scores to index cognitive and emotional components of empathy, respectively.

### Spatial tasks

#### Line Bisection

Nine 16 cm horizontal lines were each presented on a single sheet of paper. Each line was centered vertically on the sheet in one of three possible horizontal locations (left aligned, right aligned, or centered). Each sheet was placed centrally in front of the subject, who was instructed to make a small mark through the center of the line. Each line was measured to find the deviation of the mark from true center, and the mean deviation was calculated. The proportional frequency of lateral errors, or *Index*, was calculated by subtracting the number of leftward errors from the number of rightward errors and dividing this difference by the total number of errors. For both of these measures, negative scores represent leftward errors.

#### Perspective-taking Task

**Figure 1:**
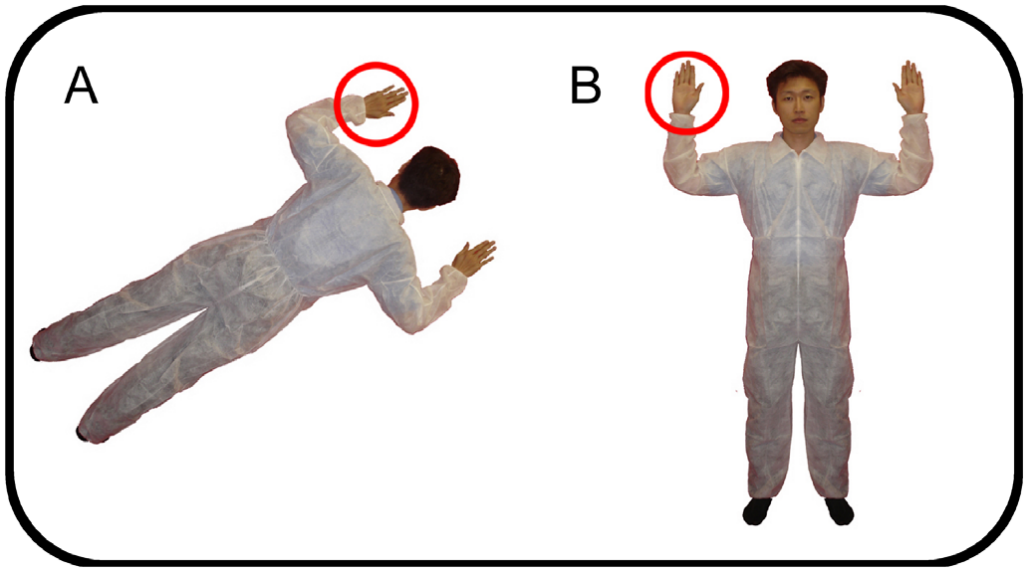
Example stimuli in the Perspective-taking Task. *Panel A.* Back-facing condition; no perspective transformation is required. *Panel B.* Front-facing condition; requires imagined self-other transformation.

We used stimuli similar to those used in previous studies of perspective-taking and mental self-other transformations A photograph of a man with his arms out to the side faced either toward or away from the participant and was rotated around the center by 0°, 60°, 120°, or 180°. Different angles of presentation is common practice in studies of imagined self-other transformations and mental rotation of objects [10], [12], [38], and were used in order to discourage participants from memorizing associations between particular stimuli and motor responses [see 11]. The stimulus figure was presented against a background of a schematic door or bed, or against a blank background as part of an exploratory analysis of whether there were differences in the efficiency of performing a self-other transformation on a figure in a supine versus upright condition. Either the right or left hand was marked by a red circle (Figure 1). Participants were asked to imagine themselves in the position of the figure on the screen and indicate whether the circled hand would be their right or left hand by pressing a key corresponding to left and right, as quickly and accurately as possible. A left judgment was indicated by the left key press and right judgment was indicated by the right key press, using their middle and index fingers. Stimulus presentation and response collection was controlled by Matlab [39].

Stimuli extended 17° degrees of visual angle horizontally and vertically and were presented in the center of the computer screen until a response was made or after a 10 s time-out period. A black fixation cross was presented during the 1000 ms intertrial interval before the next trial could begin. Response hand was counterbalanced across subjects and approximately balanced within genders (males: 11 left hand responders; females: 9 left hand responders). The mapping of finger onto left or right hand response was dependent on response hand. When using the left hand, left and right hand responses mapped onto the middle and index finger, respectively; mappings of finger onto response was reversed when using the right hand to respond. Such finger-mapping is commonplace in work on spatial stimulus-response compatibilities [40]–[42]. The experiment consisted of 384 total trials, divided into four blocks, which consisted of 8 presentations of each stimulus type in a randomized order. Trials in which the subject did not respond within the 10 s time-out period were excluded from further analysis.

Number of errors and response times of correct trials were the dependent variables of interest. The *Perspective-taking RT* was defined as the relative increase in RT for making a hand judgment for front versus back facing figures using the formula [(mean Front RT-mean Back RT)/mean Back RT], using only correct trials. Increased Perspective-taking RT indicated a relative increase in time needed to perform an imagined perspective change.

Following the experiment, subjects were asked to report the strategy they used to perform the task, and written reports were categorized by whether the participant used a body-centered strategy or not. Written strategies that were too ambiguous to rate were excluded from analysis. They were also asked to rate how strongly they imagined themselves in the perspective of the figure on the computer screen on a scale from 0 (not at all) to 6 (very strongly).

### Testing procedure

The above tasks were conducted as part of a larger battery of visuo-spatial and personality measures. The task order was counterbalanced across subjects.

### Data analysis

Separate repeated measures ANOVAs were conducted on mean RT and error rate in the Perspective-taking task, with gender entered as a between-subject variable and background, perspective, and angle of rotation entered as within-subject variables. Shapiro-Wilk statistics indicated that Perspective-taking RT, line bisection mean deviation and index scores, and IRT PT subscale scores were distributed normally (all p-values>.10). However, scores on the IRI EC subscale were not normally distributed (W = 0.94, p = .02). For all normally distributed variables, pairwise gender comparisons were assessed using independent two-sample t-tests. Independent one-sample t-tests were used to compare the frequency of lateral errors and Perspective-taking RT to a hypothesized mean of 0. Pearson correlation coefficients were calculated to evaluate the relationship between continuous variables, both within and across genders. For the IRI EC subscale, gender comparisons were assessed with the Wilcoxon test, and Spearman rank-correlation coefficients were used to evaluate the relationship between scores on this subscale and other continuous measures. Pearson Chi-squared tests were used to examine the significance of gender on differences in self-reported strategies. All tests were two-tailed, except where otherwise noted, and the alpha level was set at 0.05. Univariate outliers were identified as those individuals who scored outside three s.d.'s from the within-gender mean and were excluded from further analyses involving those measures.

## Results

### Self-reported empathy scale

Means for the IRI PT and EC subscales are displayed in Table 1. One-tailed tests were used to compare scores across males and females, as previous research has found females score higher on all of the IRI subscales [35]. No gender difference was observed on the PT subscale (t(38) = .07, p = .47), but the mean IRI EC subscale score was higher for females (Z = 1.69, p = .05).

**Table 1:** Mean scores for self-reported empathy and spatial tasks, both collapsed and split by gender (mean±s.d.).

	Males (n = 21)	Females (n = 19)	All subjects
Interpersonal Reactivity Index			
Perspective-Taking	17.71±3.02	17.79±4.22	17.75±3.59
Empathic Concern*	18.33±4.13	20.84±3.82	19.53±4.13
Spatial Tasks			
Line Bisection: IndexScore	−0.18±0.53	−0.06±0.56	−0.12±0.54
Line Bisection: MeanDeviation	−0.74±2.22	−0.28±2.51	−0.52±2.34
Perspective-taking RT^a^	0.25±0.08	0.21±0.09	0.23±0.09

a One male subject was removed because his RT was above three standard deviations from the mean Perspective-taking RT.

* Gender difference, p<.05.

### Spatial tasks

#### Line Bisection

The mean deviation and index score are displayed in Table 1. No gender differences were observed using either measure (mean deviation: t(38) = 0.62, p = .54; index score: t(38) = 0.66, p = .51). Since we had an a priori hypothesis of a significant leftward bias on the line bisection task based on extensive previous literature, a one-tailed one-sample independent t-test was conducted to compare the mean deviation and index score to 0. As there was no gender difference, we only examined pseudoneglect collapsed across genders. There was a trend towards a leftward bias using both the mean deviation (t(39) = 1.44, p = .08) and index scores (t(39) = 1.41, p = .08). Although this effect did not reach significance, the mean leftward deviation (0.52 mm) is comparable to that found in previous studies [e.g. 32].

#### Perspective-taking Task

We were primarily interested in effects of gender and perspective of the stimulus figure (front- versus back-facing).

### Errors

There was a significant effect of perspective on error rate (F(1,38) = 20.4, p<.0001), although error rates for both conditions were low (front-facing: 3.5±3.3%, back-facing: 1.6±1.9%). Full ANOVA (Table S1) and means (Table S2) tables for error rate are included as supporting information. Pearson correlation coefficients were calculated to investigate the relationship between RTs and error rates in both the front- and back-facing conditions. In the back-facing condition, there was no significant relationship between RT and error rate (r = .25, p = .12). In the front-facing condition mean RT and error rate were significantly positively correlated (r = .44, p = .004); higher error rates were associated with longer RTs. Error rates and RTs being higher in the more difficult front-facing condition and RTs and error rates being positively correlated in the front-facing condition support the notion that results cannot be explained by a speed-accuracy tradeoff.

### RT

There was a robust effect of perspective on mean RT (F(1,38) = 128.5, p<.0001), with slower performance in the front-facing (1183±346 ms) versus back-facing condition (948±253 ms). There were no main effects of gender, nor any gender interaction effects. Full ANOVA (Table S1) and means (Table S2) tables for RTs are included as supporting information.

### Perspective-taking RT

One male participant was excluded from this analysis because of Perspective-taking RT greater than three s.d.'s from the gender mean. Mean scores are displayed for the remaining 39 subjects in Table 1. No gender differences were observed for Perspective-taking RT (t(37) = 1.38, p = .17), so we collapsed across gender to compare Perspective-taking RT to zero. A score of zero would indicate that no additional time was needed to make a handedness judgment when an imagined change in perspective was required. Perspective-taking RT was significantly greater than zero (t(38) = 16.98, p<.0001), and this pattern was observed for all subjects. There were no differences in Perspective-taking RT across response hands for either males (t(18) = .28, p = .78) or females (t(17) = 0.99, p = .34). Although there was no effect of gender on Perspective-taking RT, females reported imagining themselves in the perspective of the figure on the screen significantly stronger than men (men: 3.65±1.90, women: 4.68±1.16; p = .05). Moreover, there was a trend for more males than females to report use a non-egocentric strategy (26.3% vs. 5.9%; χ^2^ (1, N = 35) = 3.19, p = .07). Three males and two females were removed from this analysis because of ambiguous reported strategies.

### Empathy-Visuo-spatial correlations

Correlations and p-values are displayed in Table 2.

**Table 2:** Correlations between empathy and spatial tasks.

	Males (n = 21)		Females (n = 19)		All subjects	
	r	p	r	p	r	p
Line Bisection Index Score						
Perspective-Taking a	0.07	.78	0.12	.62	0.09	.55
Empathic Concern	0.48*	.04	0.61**	.003	0.55**	.0002
Perspective-taking RT						
Perspective-Taking a	−0.04	.86	0.27	.26	0.14	.38
Empathic Concern a	−0.17	.47	0.70**	.0008	0.22	.18

a One male subject was removed because his RT was above three standard deviations from the mean Perspective-taking RT.

* p<.05.

** p<.01.

#### Line bisection and empathy correlations

**Figure 2:**
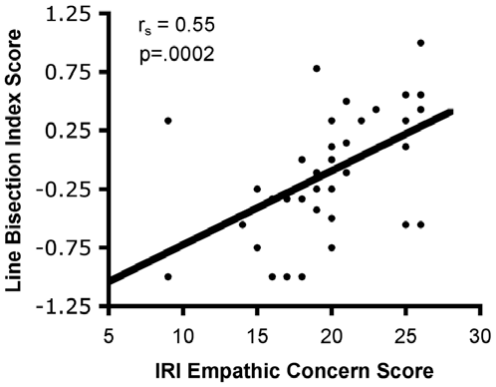
Relationship between empathic concern scores and line bisection index scores. Negative index scores indicate a leftward bias on the line bisection task.

Since the line bisection mean deviation and index score were tightly correlated (r = .90, p<.0001), only the index score was used to examine the association between self-reported empathy subscales and line bisection. Collapsed across genders, scores on the EC subscale of the IRI were positively correlated with line bisection index scores (Figure 2; r_s_ = .55, p = .0002), such that higher empathic concern scores were associated with more frequent rightward line bisections, and this correlation was significant in both males (r_s_ = .61, p = .003) and females (r_s_ = .48, p = .04). However, scores on the IRI PT subscale did not correlate with line bisection scores, either across (r = 0.10, p = .55) or within genders (males: r = .07, p = .78; females: r = 0.12, p = .62).

#### Perspective-taking RT and empathy correlations

**Figure 3:**
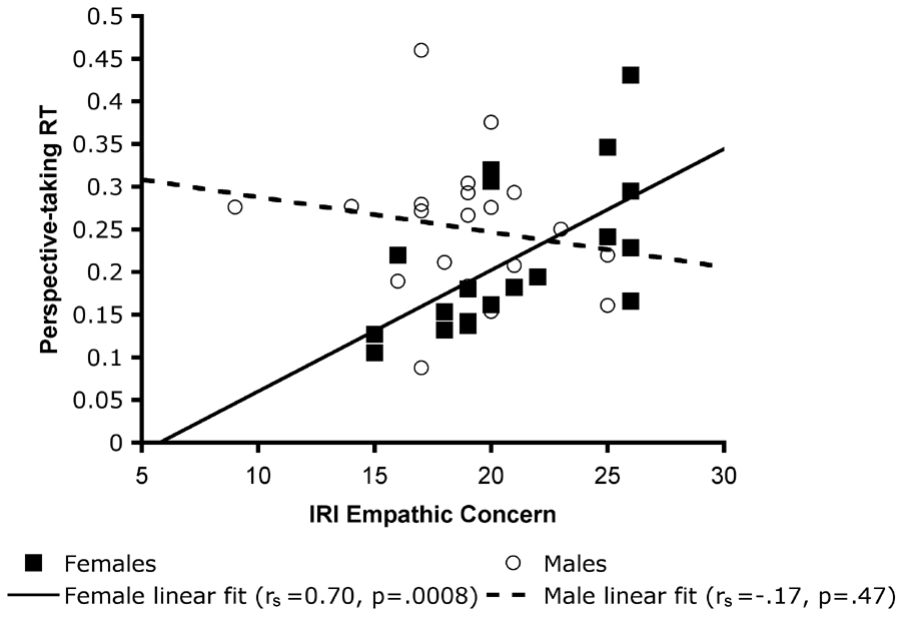
Relationship between empathic concern scores and Perspective-taking RT, (mean Front RT-mean Back RT)/mean Back RT.

Again, one male was excluded from analysis for being an outlier on the Perspective-taking RT. Collapsed across gender, there was no significant correlation between the IRI EC subscale and Perspective-taking RT (r_s_ = 0.22, p = .18). Examining the correlations by gender revealed a significant correlation in women (r_s_ = 0.70, p = .0008), but not men (r_s_ = −0.17, p = .47). That is, in women only, longer RTs needed to perform a perspective transformation were associated with increased self-reported empathic concern (Figure 3).

This result was contrary to our initial hypothesis, but an alternative and plausible account might be that in women, a decrease in Perspective-taking RT reflects an overly blurred distinction between self and other. That is, less time needed to make an imagined self-other physical transformation could be related to less distinct mental representations of self and other. This blurred self-other distinction could be reflected in susceptibility to emotion contagion, which refers to incorporating the affective state of another person without being aware that it is not your own feeling—essentially, not maintaining a self-other distinction. The Personal Distress scale of the IRI is related to measures of emotion contagion [43]. Post-hoc analyses revealed that the PD scale was marginally associated with speed of a perspective transformation in females (r_s_ = −0.39, p = .10), but not males (r_s_ = 0.13, p = .58). Moreover, in females, but not males, the IRI PD scale was negatively correlated with the IRI EC scale (females: r_s_ = −0.58, p = .01; males: r_s_ = −0.35, p = .14). Thus, in females, more interpersonal anxiety due to distress in another was significantly associated with less empathic concern and marginally related to faster Perspective-taking RT.

There was no significant correlation between the IRI PT subscale and Perspective-taking RT either across (r = 0.14, p = .38) or within genders (males: r = −0.04, p = .86; females: r = 0.27, p = .26).

## Discussion

We examined the relationship between visuo-spatial processing, specifically imagined self-other transformations and biases in spatial attention, and self-reported empathy in healthy young individuals. Specifically, we tested two hypotheses, which are addressed here in turn.

First, we expected participants' efficiency of self-other transformations (“embodied perspective-taking”) to be correlated with increased self-reported empathy, given its face validity and evidence for common brain regions involved in both functions. Contrary to this expectation, we found that, in the women, speed of visuo-spatial self-other transformations was associated with *decreased* self-reported empathic concern. Although purely speculative at this point, one possible reason for this counterintuitive finding is that self-other perspective changes reportedly increase self-referential processing [44]. Ames, et al. [44] found that adopting the cognitive perspective of another individual increased activity in medial frontal regions associated with introspection. In women, empathic concern was *negatively* associated with scores on the Personal Distress scale of the IRI. Again, this scale is related to measures of susceptibility to emotion contagion [43] and a putative decrease in a self-other distinction [45]. This finding of an association between higher empathic concern and lower personal distress is consistent with prior work. Hoffman [46] proposed a theory in which perspective-taking and sympathy are *negatively* related to feelings of personal distress when observing another individual in distress, whereby one cannot differentiate self from other. Moreover, Davis [37] found that the IRI PD scale, which measures anxiety in tense interpersonal settings, was negatively correlated with other empathy measures. It is possible that more efficient visuo-spatial self-other transformations were associated with an increased tendency towards self-referential processing, and thus a decrease in trait empathic concern, which is associated with prosocial, other-oriented behaviors. This argument is bolstered by our finding of a marginal, albeit nonsignificant, decrease in Perspective-taking RT with increasing PD scores in women. That is, in women, increased scores of personal distress when observing another in distress tended to be associated with faster self-other transformations.

Although, to our knowledge, there are no studies that directly address the relationship between self-referential processing and speed of visuo-spatial perspective-taking, there are some bodies of research that speak indirectly to this idea. Research investigating vantage point taken in episodic memories has shown that asking people to focus on objective circumstances associated with a particular event led them to recall the event from an observer's perspective, whereas asking individuals to focus on the feelings associated with the event leads to recollection of the event from a first-person perspective [47]. Likewise, being instructed to recall a particular event from an observer's perspective leads to recollection of more concrete information about the event, whereas instructions to recall the event from a first-person perspective led to more self-oriented descriptions of affective reactions and physical sensations [48].

Moreover, work by Kühnen and Oyserman [49], found that manipulation of self-focus improved speed and accuracy on tasks that were congruent with the primed self focus. On the other hand, one might also expect interpersonal anxiety to hinder the efficiency of perspective transformations given the literature on anxiety and cognitive performance [50]. To sum, it is possible that increased RT on the visuo-spatial perspective-taking task may reflect a stronger self-other distinction. However, future work with a fuller range of Personal Distress scores is needed to elucidate the nature of this relationship.

There are several possible reasons why this relationship between efficiency of imagined self-other transformations and trait empathic concern was observed in women, but not men. Firstly, women reported higher empathic concern, consistent with many previous studies of gender differences in empathy [5]. Moreover, upon debriefing, women reported imagining themselves in the perspective of the figure on the screen more strongly than men, and there was a trend for a greater proportion of women to report using a body-centered strategy. Self-report data indicates that they might have been using more of an egocentric strategy on the task. This interpretation is consistent with data indicating that men are more likely to use an object-based strategy in a spatial perspective-taking task, and women are likely to consistently employ an egocentric strategy [14]. Schulte-Rüther et al. [51] suggest that, based on recent neuroimaging work, males and females may use different cognitive and emotional processes for empathic responding which may lead to gender differences in empathy.

It is unclear why Perspective-taking RT was not related to the PT subscale of the IRI, which measures more cognitive aspects of empathy. A recent fMRI study might speak to these findings. Nummenmaa, et al. [52] found that emotional versus cognitive empathy was associated with increases in neural networks associated with body perception. Future neuroimaging work could help clarify the unique relationship between cognitive versus affective empathy and visuo-spatial perspective-taking.

Our second hypothesis was that increased self-report empathy scores would be associated with a leftward attentional bias, given the literature on right hemispheric involvement in empathy and related altruistic behaviors. Our prediction was not confirmed. Most strikingly, we found a robust relationship, in women and men, between *rightward* deviations in line bisection and self-reported empathic concern. This could indicate an association of increased empathic concern with either increased left hemispheric activation or decreased right hemispheric activation, or a hemispheric activation imbalance. On second consideration, this finding is not entirely inconsistent with the existing literature. Rightward bias on the line bisection task was only associated with the empathic concern subscale of the IRI, not the more cognitive perspective-taking subscale. The empathic concern subscale was developed to measure more prosocial aspects of empathy [37], and, as described in the introduction, there is evidence for a leading role of the left hemisphere in prosocial motivations [see 23].

It is interesting to note that left hemisphere activation has long been associated with positive emotion and approach behavior, whereas right hemisphere activation has been linked to negative emotion and withdrawal behavior [53]–[55]. More specifically, anxious arousal and threat-related information have been linked to the right posterior activation [53], [56]. The observed correlation between empathic concern and rightward attentional bias may be interpreted in this context; one might hypothesize that empathic concern is not possible when one is feeling threatened. However, data on hemispheric asymmetry of emotional functions must be interpreted with caution as most studies measure *relative* activity of the two hemispheres (e.g. left hemisphere>right hemisphere).

Another possible explanation is related to the laterality of emotional facial expressions. It has been found that the left side of the face is more emotionally expressive, possibly because of the role of the right hemisphere in emotion perception and expression [57]. The left side of the face would be in the right visual field of an observer, and, possibly, a rightward bias in spatial attention may enhance one's ability to perceive emotions in other people. Specifically, it is distressed emotional cues that reportedly elicit empathic responding [see 7].

Although, to our knowledge, there has not been much evidence for changes in emotional empathy in individuals with right parietal damage resulting in unilateral neglect, the existing literature on Williams Syndrome may shed some light on this issue. Williams Syndrome is a developmental syndrome characterized by low intellectual functioning, heightened sociability and empathy, a relative strength in language, and profound visuo-spatial impairments [58]. More in-depth studies of empathy in Williams syndrome have suggested that only the affective, not cognitive components, of empathy are spared or superior [59]. Interestingly, the spatial impairments found in Williams syndrome have been likened to those with right hemisphere damage [60]. Thus, work in developmental psychopathology provides some evidence for a link between emotional empathy and deficits in right hemisphere mediated visuo-spatial functions.

In conclusion, we found that one facet of trait-level empathy, empathic concern, was associated with performance in two visuo-spatial tasks. First, efficiency in performing an imagined self-other visuo-spatial transformation was associated, in women but not men, with decreased self-reported empathic concern. Second, increased rightward biases in line bisection were associated with increased self-reported empathic concern, pointing to greater left compared to right hemisphere mediation of this kind of empathy.

As we do not have substantial neuroimaging or lesion data that speak to these results, we acknowledge that these links between visuo-spatial perspective-taking, biases in spatial attention, and empathy are somewhat tenuous, and our conclusions are only speculative. These are complex issues, but with these data, we can offer a hypothesis for future studies. Although further research is needed to replicate and refine these results, the paradigms employed here provide a unique approach to examining relationships between basic cognitive functions and complex social constructs.

## Supporting Information

Table S1 Summary table for analyses of variance of mean response time and accuracy in the Perspective-taking task.
(0.10 MB DOC)

Table S2 Mean RT, in seconds, and error rate across conditions in the Perspective-taking task.
(0.05 MB DOC)

## References

[1] Eliot TS (1916). Knowledge and Experience in the Philosophy of F.H. Bradley.

[2] Marshall JC, Fink GR (2001). Spatial cognition: where we were and where we are.. Neuroimage.

[3] Iacoboni M, Dapretto M (2006). The mirror neuron system and the consequences of its dysfunction.. Nat Rev Neurosci.

[4] Decety J, Lamm C (2007). The role of the right temporoparietal junction in social interaction: how low-level computational processes contribute to meta-cognition.. Neuroscientist.

[5] Hoffman ML (1977). Sex differences in empathy and related behaviors.. Psychol Bull.

[6] Voyer D, Voyer S, Bryden MP (1995). Magnitude of sex differences in spatial abilities: a meta-analysis and consideration of critical variables.. Psychol Bull.

[7] Preston SD, de Waal FB (2002). Empathy: Its ultimate and proximate bases.. Behav Brain Sci :.

[8] Gallese V (2003). The manifold nature of interpersonal relations: the quest for a common mechanism.. Philos Trans R Soc Lond B Biol Sci.

[9] Amorim MA (2003). “What is my avatar seeing?” The coordination of “out-of-body” and “embodied” perspectives for scene recognition across views.. Visual Cognition.

[10] Parsons LM (1987). Imagined spatial transformation of one's body.. J Exp Psychol Gen.

[11] Zacks J, Rypma B, Gabrieli JD, Tversky B, Glover GH (1999). Imagined transformations of bodies: an fMRI investigation.. Neuropsychologia.

[12] Shepard RN, Metzler J (1971). Mental rotation of three-dimensional objects.. Science.

[13] Vogeley K, Fink GR (2003). Neural correlates of the first-person-perspective.. Trends Cogn Sci.

[14] Kaiser S, Walther S, Nennig E, Kronmuller K, Mundt C (2008). Gender-specific strategy use and neural correlates in a spatial perspective taking task.. Neuropsychologia.

[15] David N, Bewernick BH, Cohen MX, Newen A, Lux S (2006). Neural representations of self versus other: visual-spatial perspective taking and agency in a virtual ball-tossing game.. J Cogn Neurosci.

[16] Creem-Regehr SH, Neil JA, Yeh HJ (2007). Neural correlates of two imagined egocentric transformations.. Neuroimage.

[17] Blanke O, Mohr C, Michel CM, Pascual-Leone A, Brugger P (2005). Linking out-of-body experience and self processing to mental own-body imagery at the temporoparietal junction.. J Neurosci.

[18] Blanke O, Landis T, Spinelli L, Seeck M (2004). Out-of-body experience and autoscopy of neurological origin.. Brain.

[19] Ruby P, Decety J (2004). How would you feel versus how do you think she would feel? A neuroimaging study of perspective-taking with social emotions.. J Cogn Neurosci.

[20] Blakemore SJ, Frith C (2003). Self-awareness and action.. Curr Opin Neurobiol.

[21] Adolphs R (2001). The neurobiology of social cognition.. Curr Opin Neurobiol.

[22] Shamay-Tsoory SG, Tomer R, Goldsher D, Berger BD, Aharon-Peretz J (2004). Impairment in cognitive and affective empathy in patients with brain lesions: anatomical and cognitive correlates.. J Clin Exp Neuropsychol.

[23] Buck R (1999). The biological affects: a typology.. Psychol Rev.

[24] Ross ED, Homan R, Buck R (1994). Differential hemispheric lateralization of primary and social emotions.. Neuropsychiatry, Neuropsychology, and Behavioural Neurology.

[25] MacLean PD, Lewis M, Haviland J (1993). Cerebral evolution of emotion.. Handbook of emotions.

[26] Gur RC, Mozley LH, Mozley PD, Resnick SM, Karp JS (1995). Sex differences in regional cerebral glucose metabolism during a resting state.. Science.

[27] Reuter-Lorenz PA, Kinsbourne M, Moscovitch M (1990). Hemispheric control of spatial attention.. Brain Cogn.

[28] Vallar G, Perani D, Jennerod M (1987). The anatomy of spatial neglect in humans.. Neurophysiological and Neuropsychological Aspects of Spatial Neglect.

[29] Robertson IH, Halligan PW (1999).

[30] Jewell G, McCourt ME (2000). Pseudoneglect: a review and meta-analysis of performance factors in line bisection tasks.. Neuropsychologia.

[31] Heilman KM, van den Abell T (1980). Right hemisphere dominance for attention: the mechanism underlying hemispheric asymmetries of inattention (neglect).. Neurology.

[32] Drake RA, Myers LR (2006). Visual attention, emotion, and action tendency: Feeling active or passive.. Cognition and Emotion.

[33] Mohr C, Bracha HS, Brugger P (2003). Magical ideation modulates spatial behavior.. J Neuropsychiatry Clin Neurosci.

[34] Chapman LJ, Chapman JP (1987). The measurement of handedness.. Brain Cogn.

[35] Davis MH (1980). A multidimensional approach to individual differences in empathy.. JSAS Catalog of Selected Documents in Psychology.

[36] Paulus C (1992). Empathie, Kompetenz und Altruismus.. http://www.unisaarland.de/fak5/ezw/abteil/motiv/paper/empathie.htm.

[37] Davis MH (1983). Measuring Individual Differences in Empathy: Evidence for a Multidimensional Approach.. Journal of Personality and Social Psychology.

[38] Parsons LM (1987). Imagined spatial transformations of one's hands and feet.. Cogn Psychol.

[39] Brainard DH (1997). The Psychophysics Toolbox.. Spat Vis.

[40] Leuthard J, Bachtold D, Brugger P (2005). Is “left” always where the thumb is right?: stimulus-response compatibilities as a function of posture and location of the responding hand.. Cogn Behav Neurol.

[41] Katz AN (1981). Spatial compatibility effects with hemifield presentation in a unimanual two-finger task.. Can J Psychol.

[42] Heister G, Ehrenstein WH, Schroeder-Heister P (1986). Spatial S-R compatibility effects with unimanual two-finger choice reactions for prone and supine hand positions.. Percept Psychophys.

[43] Doherty RW (1997). The Emotional Contagion Scale: A Measure of Individual Differences.. Journal of Nonverbal Behavior.

[44] Ames DL, Jenkins AC, Banaji MR, Mitchell JP (2008). Taking another person's perspective increases self-referential neural processing.. Psychol Sci.

[45] de Vignemont F, Singer T (2006). The empathic brain: how, when and why?. Trends Cogn Sci.

[46] Hoffman ML (1977). Empathy, its development and prosocial implications.. Nebr Symp Motiv.

[47] Nigro G, Neisser U (1983). Point of View in Personal Memories.. Cognitive Psychology.

[48] McIsaac HK, Eich E (2002). Vantage point in episodic memory.. Psychon Bull Rev.

[49] Kühnen U, Oyserman D (2002). Thinking about the self influences thinking in general: cognitive consequences of salient self-concept.. Journal of Experimental Social Psychology.

[50] Eysenck MW, Calvo MG (1992). Anxiety and performance: The processing efficiency theory.. Cognition and Emotion.

[51] Schulte-Ruther M, Markowitsch HJ, Shah NJ, Fink GR, Piefke M (2008). Gender differences in brain networks supporting empathy.. Neuroimage.

[52] Nummenmaa L, Hirvonen J, Parkkola R, Hietanen JK (2008).

[53] Heller W, Nitschke JB, Miller G (1998). Lateralization in Emotion and Emotional Disorders.. Current Directions in Psychological Science.

[54] Heller W (1993). Neuropsychological mechanisms of individual differences in emotion, personality, and arousal.. Neuropsychology.

[55] Davidson R, Davidson RJ, Hugdahl K (1995). Cerebral asymmetry, emotion and affective style.. Brain Asymmetry.

[56] Compton RJ, Heller W, Banich MT, Palmieri PA, Miller GA (2000). Responding to threat: Effects of hemispheric asymmetry and interhemispheric division of input.. Neuropsychology.

[57] Sackeim HA, Gur RC, Saucy MC (1978). Emotions are expressed more intensely on the left side of the face.. Science.

[58] Morris CA, Mervis C, Goldstein S, Reynolds C (1999). Williams Syndrome.. Handbook of neurodevelopmental and genetic disorders in children.

[59] Tager-Flusberg H, Sullivan K (2000). A componential view of theory of mind: evidence from Williams syndrome.. Cognition.

[60] Bellugi U, Lichtenberger L, Jones W, Lai Z, St George M (2000). I. The neurocognitive profile of Williams Syndrome: a complex pattern of strengths and weaknesses.. J Cogn Neurosci.

