# Supplementary material for: Exploring Empathic Space: Correlates of Perspective Transformation Ability and Biases in Spatial Attention
Source: PLoS One. 2009 Jun 10;4(6):e5864. doi: 10.1371/journal.pone.0005864 (PMC2688758; doi:10.1371/journal.pone.0005864)
Supplement: Table S1 — Summary table for analyses of variance of mean response time and accuracy in the Perspective-taking task. (0.10 MB DOC) [file pone.0005864.s001.doc]

Table S1. Summary table for analyses of variance of mean response time and accuracy in the Perspective-taking task.

| **Measure** | **Effect** | **F** | **df** | **MSE** | **p** |
| --- | --- | --- | --- | --- | --- |
| **RT** | Gender | 0.45 | 1,38 | 2.16 | 0.50 |
|  | Background | 4.71 | 2,76 | 0.02 | 0.01 |
|  | Gender*Background | 1.34 | 2,76 | 0.02 | 0.27 |
|  | Perspective | 128.51 | 1,38 | 0.10 | <.0001 |
|  | Gender*Perspective | 3.41 | 1,38 | 0.10 | 0.07 |
|  | Background*Perspective | 21.48 | 2,76 | 0.02 | <.0001 |
|  | Gender*Background*Perspective | 0.27 | 2,76 | 0.02 | 0.77 |
|  | Rotation Angle | 76.88 | 3,114 | 0.08 | <.0001 |
|  | Gender*Rotation Angle | 1.28 | 3,114 | 0.08 | 0.29 |
|  | Background*Rotation Angle | 3.23 | 6,228 | 0.02 | 0.005 |
|  | Gender*Background*Rotation Angle | 0.85 | 6,228 | 0.02 | 0.53 |
|  | Perspective*Rotation Angle | 26.88 | 3,114 | 0.06 | <.0001 |
|  | Gender*Perspective*Rotation Angle | 0.32 | 3,114 | 0.06 | 0.81 |
|  | Background*Perspective*Rotation Angle | 3.54 | 6,228 | 0.01 | 0.002 |
|  | Gender*Background*Perspective*Rotation Angle | 1.71 | 6,228 | 0.01 | 0.12 |
| **Accuracy** | Gender | 0.01 | 1,38 | 141.44 | 0.92 |
|  | Background | 0.16 | 2,76 | 12.51 | 0.86 |
|  | Gender*Background | 0.35 | 2,76 | 12.51 | 0.70 |
|  | Perspective | 20.43 | 1,38 | 39.53 | <.0001 |
|  | Gender*Perspective | 0.37 | 1,38 | 39.53 | 0.55 |
|  | Background*Perspective | 0.43 | 2,76 | 13.03 | 0.65 |
|  | Gender*Background*Perspective | 1.59 | 2,76 | 13.03 | 0.21 |
|  | Rotation Angle | 9.65 | 3,114 | 46.92 | <.0001 |
|  | Gender*Rotation Angle | 0.16 | 3,114 | 46.92 | 0.92 |
|  | Background*Rotation Angle | 0.24 | 6,228 | 13.44 | 0.96 |
|  | Gender*Background*Rotation Angle | 1.15 | 6,228 | 13.44 | 0.34 |
|  | Perspective*Rotation Angle | 1.30 | 3,114 | 34.93 | 0.28 |
|  | Gender*Perspective*Rotation Angle | 1.08 | 3,114 | 34.93 | 0.36 |
|  | Background*Perspective*Rotation Angle | 2.23 | 6,228 | 12.22 | 0.04 |
|  | Gender*Background*Perspective*Rotation Angle | 0.97 | 6,228 | 12.22 | 0.45 |
