# Supplementary material for: Exploring Empathic Space: Correlates of Perspective Transformation Ability and Biases in Spatial Attention
Source: PLoS One. 2009 Jun 10;4(6):e5864. doi: 10.1371/journal.pone.0005864 (PMC2688758; doi:10.1371/journal.pone.0005864)
Supplement: Table S2 — Mean RT, in seconds, and error rate across conditions in the Perspective-taking task. (0.05 MB DOC) [file pone.0005864.s002.doc]

Table S2. Mean RT, in seconds, and error rate across conditions in the Perspective-taking task.

|  |  | **No Background** | | | | **Supine Background** | | | | **Upright Background** | | | |
| --- | --- | --- | --- | --- | --- | --- | --- | --- | --- | --- | --- | --- | --- |
|  |  | **0°** | **60°** | **120°** | **180°** | **0°** | **60°** | **120°** | **180°** | **0°** | **60°** | **120°** | **180°** |
| **RT (s)** | **back** | 0.70 | 0.75 | 0.89 | 1.23 | 0.77 | 0.91 | 0.93 | 1.30 | 0.79 | 0.89 | 0.92 | 1.32 |
|  | **front** | 1.19 | 1.09 | 1.15 | 1.40 | 1.23 | 1.08 | 1.07 | 1.34 | 1.16 | 1.14 | 1.10 | 1.29 |
| **% Error** | **back** | 0.31 | 0.47 | 1.56 | 3.91 | 0.31 | 0.63 | 2.81 | 3.75 | 0.47 | 0.63 | 1.09 | 3.75 |
|  | **front** | 3.13 | 2.50 | 3.28 | 5.16 | 3.75 | 2.34 | 1.56 | 6.09 | 3.13 | 2.66 | 3.44 | 4.84 |
